# P-1526. *In vitro* Activity of Ceftobiprole Against Prominent Methicillin-resistant *Staphylococcus aureus* Clonal Groups Collected Through the SENTRY Antimicrobial Surveillance Program

**DOI:** 10.1093/ofid/ofae631.1695

**Published:** 2025-01-29

**Authors:** Rodrigo E Mendes, Hank Kimbrough, Joshua Maher, Jennifer Smart, Mark E Jones, Mariana Castanheira, Helio S Sader

**Affiliations:** JMI Laboratories, North Liberty, Iowa; Element Materials Technology/Jones Microbiology Institute, North Liberty, Iowa; Element Materials Technology/Jones Microbiology Institute, North Liberty, Iowa; Basilea Pharmaceutica International Ltd, Allschwil, Basel-Landschaft, Switzerland; Basilea Pharmaceutica International Ltd., Allschwil, Switzerland, Allschwil, Basel-Landschaft, Switzerland; JMI Laboratories, North Liberty, Iowa; JMI Laboratories, North Liberty, Iowa

## Abstract

**Background:**

Ceftobiprole (BPR), an advanced generation cephalosporin, was recently (April 2024) approved by the US FDA for treating *Staphylococcus aureus* bacteremia (SAB), community-acquired bacterial pneumonia (CABP), and acute bacterial skin and skin structure infections (ABSSSI). This study examined the activity of BPR and the comparator agent ceftaroline (CPT) against a contemporary (2018-2019) collection of methicillin-resistant *S. aureus* (MRSA) isolates from major pandemic lineages.

Genomic features and susceptibility profile of MRSA isolates stratified by clonal group and subgroups.

**Figure:**
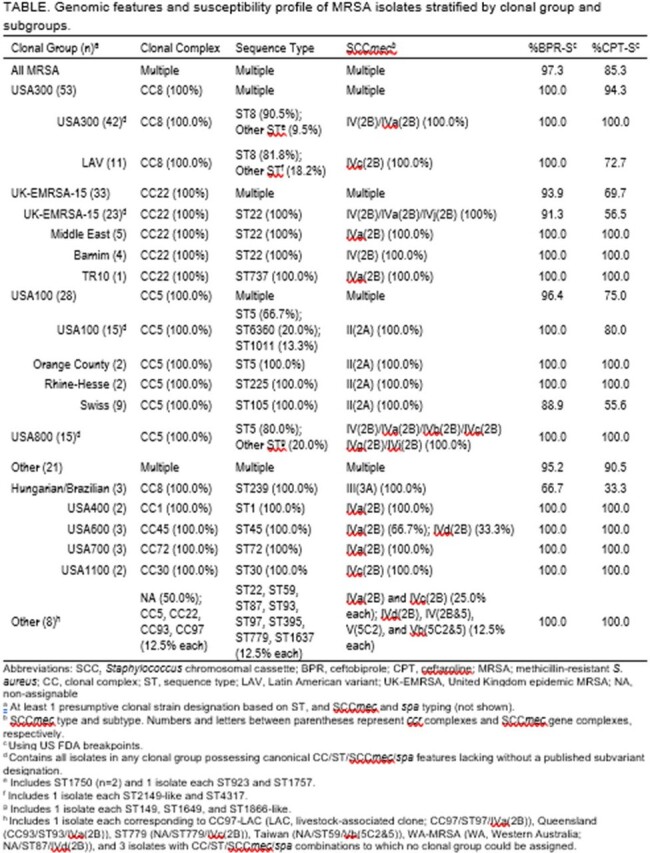

**Methods:**

150 MRSA collected from 38 hospitals in 18 countries were susceptibility (S) tested against BPR and CPT using CLSI broth microdilution methods; FDA breakpoints were applied. Total genomic DNA was extracted and sequenced. Assembled genomes were used to determine multi-locus sequence type (ST), clonal complex (CC), *spa* (not shown) and SCC*mec* types; results of typing methods were used to assign clonal group (CG).

**Results:**

Twenty-four CGs, including subdivisions, were identified; 3 isolates could not be assigned a CG. Prominent CGs were USA300 (n=53, 35.3%), UK-EMRSA-15 (n=33, 22.0%), USA100 (n=28, 18.7%), and USA800 (n=15, 10.0%); rare CGs (≤ 3 isolates; n=21) totaled 14.0% (TABLE). Against all MRSA, 97.3% were BPR-S and 85.3% were CPT-S. BPR was more active than CPT across CGs and subdivisons: USA300, 100.0% BPR-S/94.3% CPT-S; UK-EMRSA-15, 93.9% BPR-S/69.7% CPT-S; USA100, 96.4% BPR-S/75.0% CPT-S; USA800, 100.0% BPR-S/CPT-S. Against rare or unknown CGs, 95.2% were BPR-S and 90.5% were CPT-S. Four (2.7%) and 22 (14.7%) isolates were BPR- and CPT-NS, respectively; all BPR-NS isolates were CPT-NS whereas 81.8% of CPT-NS isolates were BPR-S. No CG feature united BPR-NS isolates (n=2 UK-EMRSA-15, CC22/ST22/*SCCmec*-IV(2B); USA100-Swiss, CC5/ST105/SCC*mec*-II(2A); Hungarian-Brazilian; CC8/ST239/SCC*mec*-III(3A)), whereas 19/22 (86.4%) CPT-NS isolates were from subgroups of UK-EMRSA-15, USA100, or Hungarian-Brazilian CGs.

**Conclusion:**

This study reports the potent activity of BPR against a global collection of MRSA from prominent pandemic lineages. The low frequency of BPR-NS isolates, particularly among highly represented CGs including UK-EMRSA and USA100, and its activity against CPT-NS isolates, support the use of BPR in the treatment of MRSA-associated SAB, ABSSSI, and CABP.

**Disclosures:**

**Rodrigo E. Mendes, PhD**, GSK: Grant/Research Support **Jennifer Smart, PhD**, Basilea Pharmaceutica International Ltd: Employee|Basilea Pharmaceutica International Ltd: Stocks/Bonds (Public Company) **Mark E. Jones, PhD**, Basilea Pharmaceutica International Ltd: Employee|Basilea Pharmaceutica International Ltd: Stocks/Bonds (Public Company)

